# Local field potential signal transmission is correlated with the fractional anisotropy measured by diffusion tractography

**DOI:** 10.1093/braincomms/fcaf336

**Published:** 2025-09-10

**Authors:** Maral Kasiri, Sumiko Abe, Rahil Soroushmojdehi, Estefania Hernandez-Martin, Seyyed Alireza Seyyed Mousavi, Terence D Sanger

**Affiliations:** Department of Biomedical Engineering, University of California, Irvine, CA 92697, USA; Department of Neurology, Children’s Health Orange County, Orange, CA 92868, USA; Department of Electrical Engineering and Computer Science, University of California, Irvine, CA 92697, USA; Department of Electrical Engineering and Computer Science, University of California, Irvine, CA 92697, USA; Department of Electrical Engineering and Computer Science, University of California, Irvine, CA 92697, USA; Department of Biomedical Engineering, University of California, Irvine, CA 92697, USA; Department of Neurology, Children’s Health Orange County, Orange, CA 92868, USA; Department of Electrical Engineering and Computer Science, University of California, Irvine, CA 92697, USA

**Keywords:** deep brain stimulation, diffusion tractography, dystonia, functional connectivity, LFP signal transmission

## Abstract

In this paper we aim to examine the correlation between diffusion tensor imaging parameters of anatomical connectivity and characteristics of signal transmission obtained from patient-specific transfer function models. Here, we focused on elucidating the correlation between structural and functional neural connectivity within a cohort of pediatric patients diagnosed with dystonia. Diffusion tractography images were obtained from 12 patients with dystonia prior to the deep brain stimulation surgery. For each patient, we processed the imaging data to estimate anatomical measures including fractional anisotropy, axial diffusivity, number of fibre tracts per unit area, and fibre tract length. After the implantation of temporary depth leads for each patient as part of their treatment plan, intracranial signals were recorded. Transfer function models of local field potential recordings and the corresponding measures of functional connectivity were computed for each patient. Linear mixed effect analysis was then employed to determine the relationship between transfer function measures and diffusion tractography parameters. Our results illustrate a positive correlation between fractional anisotropy, AD, and intrinsic neural transmission measures, representing amplification and spread of intrinsic neural oscillations, obtained from the transfer functions models. However, no significant correlation was found between the functional connectivity and number of fibre tracts or fibre lengths. Our findings suggest that white matter integrity, as measured by fractional anisotropy and AD, can potentially reflect the amplification and spread of intrinsic brain signals throughout the network. This study underscores the significant relationship between structural and functional connectivity, offering valuable insights into propagation of neural activity in the brain network and potential implications for optimizing non-invasive treatments and planning for neurological disorders.

## Teaser

Kasiri *et al*. investigated the correlation between anatomical connectivity from diffusion tractography imaging and functional signal transmission in pediatric dystonia. The study found that white matter integrity and AD of tracts are significantly correlated with neural signal propagation and amplification, highlighting implications for treatment and deep brain stimulation optimization, and understanding structural-functional connectivity relationship in the brain network.

## Graphical Abstract

**Graphical Abstract:**
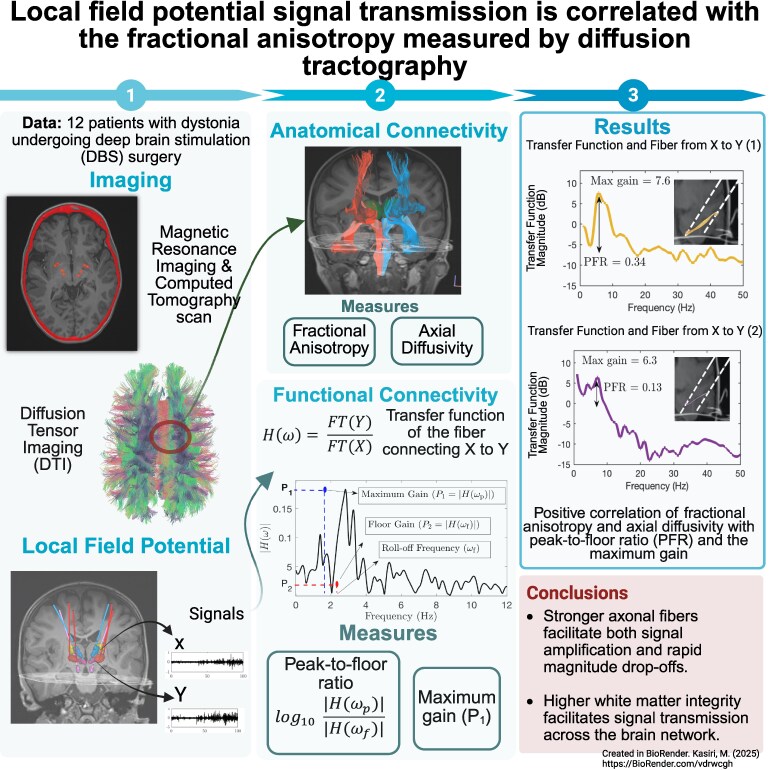

## Introduction

Advancements in neuroimaging techniques have fundamentally changed our understanding of brain functional and anatomical connectivity.^1,2^ Diffusion Tensor Imaging (DTI), as an advanced magnetic resonance imaging (MRI) modality, enables us to visualize white matter tracts that connect cortical and subcortical structures by measuring the motion of water molecules and provide us with valuable insights into the structural connectivity of the brain. With its capacity to reveal complex details of brain micro-structure, DTI plays a significant role in optimizing procedures like deep brain stimulation (DBS).^3-11^ DBS procedure can be finely tuned by utilizing DTI to precisely map neural pathways and understand microstructural connections, promising improved treatment protocols and outcomes for patients suffering from neurological disorders.^5^

Studying the relationship between structural and functional connectivity is an important domain of research for understanding the brain as a complex network of interconnected regions.^12-16^ Previous studies have explored the complex relationships and communication patterns between different brain areas in various neurological disorders. For example, such analysis in epilepsy helps to map the seizure network by exploring the relationships between the structural and functional networks responsible for conduction of epileptic activity.^15^ Moreover, such studies have been conducted on healthy subjects to find the correlation between DTI measures and resting state functional MRI (fMRI).^17^ Despite all the research endeavors that provide invaluable insights into the interaction between structural and functional connectivity, the relationship between the two for transmission of non-epileptic brain signals remains unclear.

Transfer functions have been widely used to study the signal transmission and physiological connectivity within brain regions.^18,19^ For example, Kamali *et al*.^19^ utilized characteristics of patient specific transfer function models of pathways in order to localize the seizure onset zones. In our previous studies, we have shown that transfer function models of deep brain regions can be used to replicate the evoked responses from DBS, informing us of existence of some relationship between the transmission of DBS pulses through neural pathways and the functional connectivity.^20^ Moreover, we have shown that the DBS evoked responses’ magnitude depends on the characteristics of fibre tracts such as fractional anisotropy (FA), axial diffusivity (Ad), and fibre length (*L*),^21^ informing us that the transmission of exogenous signals is predictable through the anatomical characteristics. Building upon these fundamental studies and the use transfer function methods, we aim to compute measures of endogenous signal transmission using patient specific transfer function models and find their relationship with structural measures provided by DTI, FA, Ad, *L*, and number of fibres per unit area (*N*), in basal ganglia and thalamic subnuclei. We hypothesize that the measures of signal amplification and transmission computed from transfer functions are positively correlated with the FA and Ad. In other words, we hypothesize that the white matter integrity of the fibres and the diffusivity of neural pathways are reflected in functional connectivity represented by the transfer function measures.

In order to do so, we recorded signals from deep brain regions in 12 patients with dystonia, who underwent DBS procedure as part of their clinical evaluation. MRI, computed tomography (CT) scans, and DTI images were acquired, and the DTI anatomical measures were calculated. Transfer functions representing each pathway for all patients were computed. We then compared the DTI measures and the transfer function signal transmission quantities using linear mixed effect model (LME). Understanding the correlation and mapping between DTI parameters that can be calculated non-invasively and characteristics of transfer function models, which require invasive measurements from deep brain regions, offers invaluable insights into the relationship of brain structure and function and results in improvement of treatment protocols, planning, and outcomes.

## Materials and methods

### Subjects

Twelve pediatric and young adult patients, diagnosed with primary or secondary dystonia by a movement disorder specialist (T.D.S), underwent a staged implantation of DBS leads,^22,23^ as part of their treatment plan. The patients were qualified for the treatment if there existed potential stimulation target(s) for improvement of dystonic symptoms and if alternative medical therapies were not improving the symptoms.^22,23^

The patients or the guardians of minors provided Health Insurance Portability and Accountability Act (HIPAA) authorization for research use of electrophysiological data before the procedure and provided written informed consent for surgical procedures conforming to standard hospital practice at Children’s Health Orange County (CHOC) or Children’s Hospital Los Angeles (CHLA), according to the declaration of Helsinki. The research use of data was approved by the institutional review board of CHOC or CHLA. The data collection, examinations, and the surgeries took place at either CHOC or CHLA, within 5 years.

Table 1 includes demographic information of all patients participated in this study.

**Table 1:** Patients demographics

Subject	Age	Gender	Etiology	Implanted regions
**S1**	15	F	CP	VA, VoaVop, VIM, GPi
**S2**	12	F	Unknown (dx CP)	VA, VoaVop, VIM, GPi
**S3**	20	M	CP	VoaVop, VIM, VPL, STN, GPi
**S4**	12	F	CP	VA, VoaVop, VIM, GPi
**S5**	13	M	H-ABC (genetic)	VoaVop, VIM, STN, GPi
**S6**	12	M	Dyskinetic CP	VA, VoaVop, VIM, STN, GPi
**S7**	13	M	GA1	VA, VoaVop, VIM, STN, GPi
**S8**	10	M	KMT2B	VA, VoaVop, VIM, PPN, STN, GPi
**S9**	8	M	GA1	VA, VoaVop, VIM, PPN, STN, GPi
**S10**	21	M	CP	VA, VoaVop, VIM, PPN, STN, SNr, GPi
**S11**	17	M	MYH2	VA, VoaVop, VIM, PPN, STN, GPi
**S12**	5	M	Dyskinetic CP	VA, VoaVop, VIM, PPN, STN, GPi

Our patients cohort includes 3 female and 9 male subjects, ages between 5 and 21 year-old. CP, Cerebral Palsy; F, female; GA1, Glutaric aciduria type 1;^24^ GPi, globus pallidus internus; KMT2B, Lysine Methyltransferase-2B^25^; M, male; PPN, pedunculopontine nucleus; STN, subthalamic nucleus; VA, ventral anterior; VIM, ventral intermedia; VoaVop, ventral oralis anterior-posterior; VPL, ventral posterolateral.

### Surgical procedure and depth recording

For each patient, up to 12 temporary AdTech MM16C depth sEEG leads (AdTech Medical Instrument Corp., Oak Creek, WI, United States) were implanted under general anesthesia in potential DBS targets depending on diagnosis and clinical symptoms using standard stereotactic procedure.^22,23^ These targets were identified based on previous studies of DBS clinical efficacy for dystonia which include subthalamic nucleus (STN), globus pallidus internus (GPi), in basal ganglia, ventral intermediate nucleus (VIM), ventral oralis anterior/posterior (VoaVop), and ventral anterior nucleus (VA), and ventral postrolateral nucleus (VPL), in thalamus, pedunculopontine nucleus (PPN) and Substantia Nigra reticulata (SNr) in the brainstem.^26,27^

The sEEG leads contain 10 high-impedance (70–90 kΩ) micro-wire electrodes (50-µm diameter) that are arranged in groups of two or three, spaced evenly around the circumference of the lead shaft in four rows. The leads were connected to Adtech Cabrio™ connectors that include a custom unity-gain preamplifier for each micro-contact. This setup allows for recordings with higher signal to noise ratio and reduced motion artifacts. All data used in this study were recorded through the high-impedance contacts, with stimulation off, and sampled at 24 kHz by a custom recording system consisting of a PZ5M 256-channel digitizer, RZ2 processor, and RS4 high speed data storage (TDT, Tucker-Davis Technologies Inc., Alachua, FL, United States). The depth electrode recording was done one day after the temporary-lead implantation. The surgical procedure and the recording setup are explained in detail in our previous publications.^21-26^

### Structural connectivity

#### Structural image acquisition and processing

For each patient, MRI and DTI were acquired before the surgery. The CT scans were acquired after the surgery. Preoperative T1-weighted structural MRI volumes and DTI were acquired using a MAGNETOM 3T MRI scanner (SIEMENS Medical Systems, Erlangen, Germany) to ensure precise anatomical localization. The settings of image acquisition were repetition time (TR) of 1800 ms, echo time (TE) of 2.25 ms, flip angle of 8°, voxel size of 1 mm^3^, and a field of view (FOV) of 240 mm^2^.

Two diffusion-weighted imaging scans with opposite phase-encoded polarity were acquired along with calibration scans to correct for magnetic field inhomogeneities,^28^ which could otherwise introduce errors in tractography reconstruction. To mitigate the phase encoding artifact in the echo-planar imaging sequence, two acquisitions were performed: one with an anterior-posterior (AP) phase encoding direction and the other with a posterior-anterior (PA) direction. The imaging parameters for these sequences were as follows: flip angle of 90°, repetition time (TR) of 9000 ms, echo time (TE) of 113 ms, voxel size of 2.5 × 2.5 × 2.5 mm^3^, FOV of 250 × 250 mm^2^, *b*-value of 1000 s/mm^2^, 6 and 30 diffusion gradient directions for PA and AP phase encoding respectively. Additionally, CT volumes were acquired using a GE scanner (GENERAL ELECTRIC Healthcare, Milwaukee, WI, USA) with a resolution of 512 × 512 × 320 mm^3^ to verify the localization of the sEEG leads.

Subsequently, the following steps were taken: (1) Pre-processing to correct distortions and motion artifacts of the DTI scans based on TOPUP and Eddy Current Correction algorithms^29^; (2) Co-registration of the DTI and postoperative CT images to the T1 anatomic volume using FLIRT tool in FMRIB Software Library (FSL v6.0.6.4)^30^; (3) Segmentation of all the subregions of the thalamus and pallidum using Freesurfer software (v7.4.1) for each subject.^31^ This segmentation is not perfect due to the asymmetrical brain regions in our patient cohort and their size; therefore, further manual adjustment was done using DSI-Studio (CHEN-09-27-2023)^8,32^; Then, a transformation matrix from MNI to individual space was computed for the pallidum and thalamus using FSL FLIRT and applied to DISTAL atlas subregions.^33^ (4) After co-registering CT images to the T1-weighted MRI, projection images^34^ were generated to accurately position sEEG leads by projecting 3D voxel values onto a 2D image, highlighting the highest attenuation values, using the DSI-Studio visualization package; and (5) Estimation of micro-contacts’ coordinates based on a linear model of the sEEG lead and assigning 3 mm diameter to the effective area.

#### Fibre tracking and DTI parameter estimation

In our study, DTI was employed to explore fibre orientations and measure diffusion properties. Fibre tracts were reconstructed using a deterministic algorithm in DSI Studio, in Q space with quantitative anisotropy > 0.1 and a turning angle of <60°, minimum fibre length of 10 mm, maximum fibre length of 200 mm, and step size of 0.5*voxel size. Seed size was chosen based on the fibre tracking region and was equal to *1000* × *size of the fibre tracking region.*^32^

Micro-contact regions within each nucleus served as both origin and target regions for fibre tracking, enabling visualization of the anatomic pathways connecting the origin and the target. Noting that all fibre tracts analyzed in this study were obtained from the pallidothalamic tracts and subthalamic fasciculus. These tracts, which connect GPi and STN to the subthalamus regions such as VA, VIM and VoaVop are the focus of our analysis. Other white matter tracts, such as the cortico-striatal tracts or thalamic radiations, were not included in our analysis.

Using the diffusion tensor, we computed several metrics to further characterize the fibre tracts linking these regions: (i) Ad, calculated as the first eigenvalue ($\lambda_{1}$) of the diffusion tensor, reflects water molecule diffusion along the principal axis of fibre tracts, indicating axonal integrity. Changes in Ad can indicate axonal degeneration or demyelination.^35^ (ii) FA, a measure derived from the variance of eigenvalues ($\lambda_{1}$, $\lambda_{2}$, $\lambda_{3}$) quantifies the degree of anisotropy in water diffusion, offering insights into the coherence and density of fibre tracts. The calculation of FA is given by Equation 1, which is scaled between 0 (isotropic diffusion) and 1 (highly anisotropic diffusion). This provides direct insight into the structural integrity of axonal fibres.^36^ (iii) Number of fibre tracts per unit area (*N*) estimates the count of individual fibre bundles connecting two regions of interest and offers information about the density of fibres within that area.^37^ (iv) Fibre tract length indicates the total length of individual fibre tracts connecting two regions of interest and provides insights into the spatial extent or reach of neural pathways.^38^


(1) $$\mathrm{FA}=\;\sqrt{\frac{1}{2}\;}\;\times \sqrt{\frac{{\left(\lambda_{1}-\lambda_{2}\right)}^{2}+{\left(\lambda_{2}-\lambda_{3}\right)}^{2}+{\left(\lambda_{3}-\lambda_{1}\right)}^{2}}{\lambda_{1}^{2}+\lambda_{2}^{2}+\lambda_{3}^{2}}}$$

### Functional connectivity

#### Electrophysiological data processing

The LFP recordings from 10 micro-contacts of each lead were notch filtered at 60 Hz and its 5 harmonics. They were then high pass filtered at 1 Hz to remove the drift. Each adjacent pair of micro-contacts recordings were subtracted from each other to capture their voltage difference (bipolar montage), which essentially removes the common noise and reveals the underlying neural activity.^39^

#### Transfer function computation

The empirical transfer function of a system is computed as the ratio of the system's output Fourier transform (FT(Y)) to the system's input Fourier transform (FT(X)) which can be estimated as:


$$H(\omega )=\;\frac{\mathrm{FT}(Y)}{\mathrm{FT}(X)}\;\approx \;\frac{\mathrm{CPSD}(X,\;Y)}{\mathrm{PSD}(X)+\;\varepsilon }$$

Where PSD(*X*) is the power spectral density of the input, CPSD (*X*, *Y*) is the cross power spectral density between input signal and output signal, with complex numbers, and ε is a regularization constant. We computed the single input-single output (SISO) transfer function model between each two bipolar recording channels. Note that the computed transfer functions are complex functions, which includes information about the phase and magnitude. Here, we only use the magnitude of these transfer functions, and not the phase shifts and delays.^19,20^

**Figure 1:**
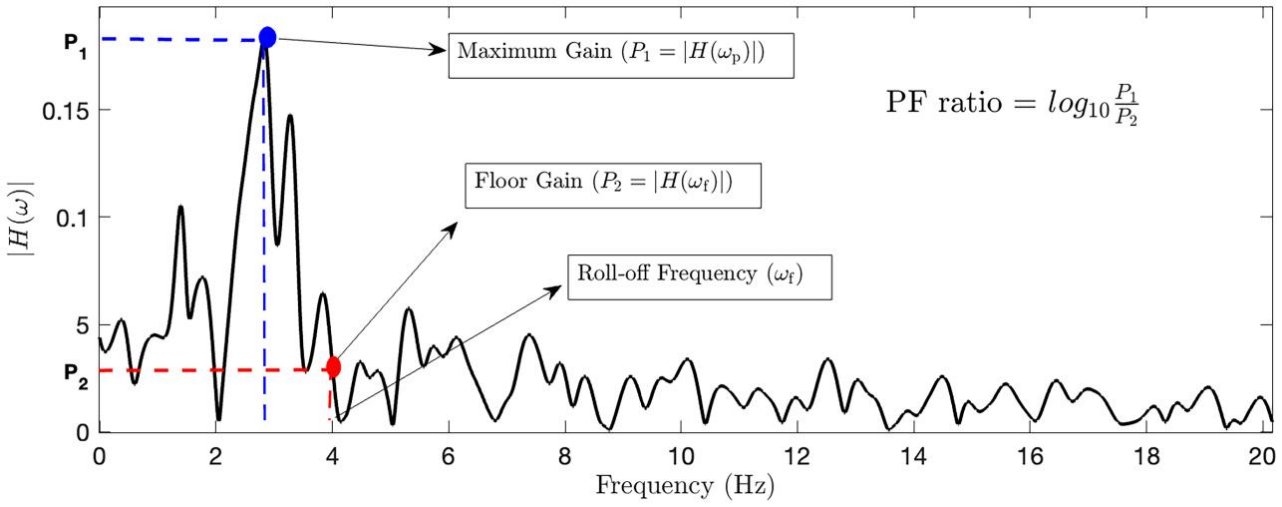
**Transfer function magnitude plot.** This figure illustrates the magnitude plot of a sample transfer function, |H(*ω*)|, from GPi to ventral oralis anterior-posterior (VoaVop), showcasing key parameters including maximum gain, floor gain, and roll-off frequency. The PF ratio is determined by calculating the ratio between peak gain (P_1_) and floor gain (P_2_).

After constructing the SISO transfer function models for each pair of channels, we computed two parameters representing the characteristics of intrinsic signal transmission from transfer function models as depicted in Fig. 1. First parameter is the peak gain or the maximum transfer function gain (P_1_ in Fig. 1), which represents the maximum level of amplification of the transmitted input signal. In other words, it is a metric that quantifies how much input signals can be amplified and spread in the network at the maximum gain frequency (*ω*_p_). The larger the gain, the more propagation and amplification of neural activity throughout the network at that frequency (*ω*_p_). The second parameter is the peak-to-floor (PF) ratio, which represents the large system responses and its fast magnitude drop-off.^19^ PF ratio is calculated as the ratio between peak of the frequency response and its magnitude at the roll-off frequency (P_2_ in Fig.1). The roll-off frequency is the boundary where the energy flowing through a system begins to drop, defined as the frequency at which the magnitude is 3 dB below the gain at frequency zero, *ω* = 0 (DC gain) or where the power drops to half the power at *ω* = 0, as P_2_ in Fig.1. For a given pathway, we calculated the frequency response magnitude of all the SISO transfer functions as quantified by the magnitude of H(*ω*). Thereafter, we computed the PF ratio as:


$$\mathrm{PF}\;\mathrm{ratio}=\mathrm{lo}g_{10}\;\frac{\left|H(\omega_{p})\right|}{\left|H(\omega_{f})\right|}$$

where *ω*  _p_ represents the frequency at which maximum gain [H(*ω*_p_)] is achieved and *ω*  _f_ is roll-off frequency and H(*ω*_f_) is the transfer function gain at the roll-off frequency. All these measures were computed for each pair of electrodes, per hemisphere.

### Statistical analysis

Here, we want to investigate whether properties of magnitude of these transfer functions correlate with the anatomical features derived from DTI data. In other words, we want to evaluate how the characteristics of these transfer functions, representing functional connectivity, are related to the anatomical connectivity in the dystonic brain and to confirm whether they contain useful information about the anatomical features of the neural fibres.

In order to evaluate the relationship between the DTI and transfer function measures, for each patient, we removed the outliers and kept the samples within 3 SD from the mean. We removed the samples for which the max gain occurs below 1.5 Hz as the data could be distorted in vicinity of 1 Hz cutoff frequency. We employed LME to model the PF ratio and the maximum gain in relation to FA, Ad, *N*, and *L* for group analysis; with Ad, *N*, FA, and *L* as the fixed effects and a random intercept for each participant to account for subject-specific variability. LME provides a robust framework for examining and assessing the strength and significance of the linear relationships between our predictors (FA, Ad, *N*, and *L*) and our outcomes of interest (maximum gain and PF ratio). Each predictor variable was chosen based on theoretical considerations and previous empirical findings suggesting their relevance to the functional connectivity.

**Figure 2:**
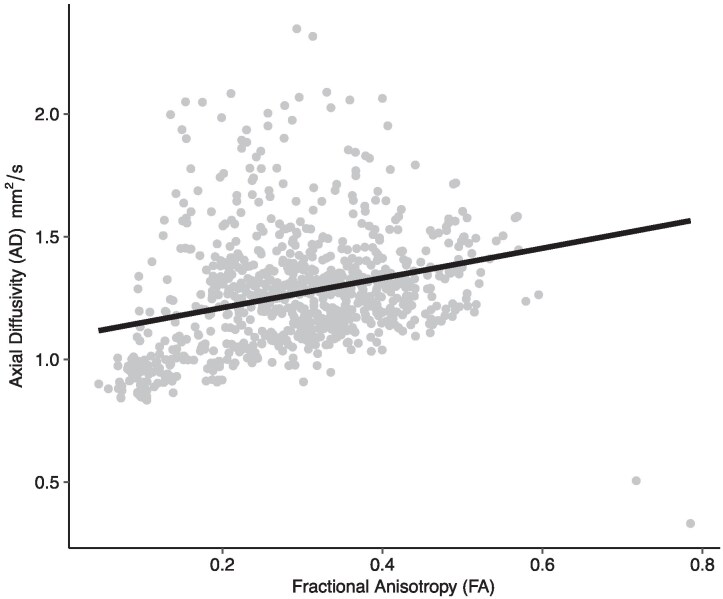
**Relationship between the FA and Ad.** To avoid the issues caused by multicollinearity, simplicity of interpretation, and to avoid redundant predictors in LME model we removed Ad from the formula and only considered FA. Each individual data point represents a tract-level observation (number of observations = 880). A linear mixed-effects model was fitted with fixed effects for FA, fibre length, and the number of fibres per unit area (*N*) and a random intercept for each participant to account for repeated measures within subjects.

One limitation of using linear models is the multicollinearity among features. Therefore, the Variance Inflation Factors (VIF) was calculated to ensure that collinearity is negligible in the models, affirming the robustness and reliability of our statistical results. After evaluating multicollinearity, we found that Ad and FA are highly positively correlated (it can also be inferred from the FA formula presented in Equation 1). Therefore, in our analysis, we considered only FA; however, in our discussion, due to their positive correlation, we addressed both parameters. Their relationship is illustrated in Fig.2.

The significance of the estimated coefficients was then evaluated by *t*-test using Satterthwaite's method with the significance threshold of 0.05. We adjusted all the *P*-values using Bonferroni method after. All the statistical analysis was done in R-studio using the lme4^40^ and emmeans^41^ packages.

## Results

**Figure 3:**
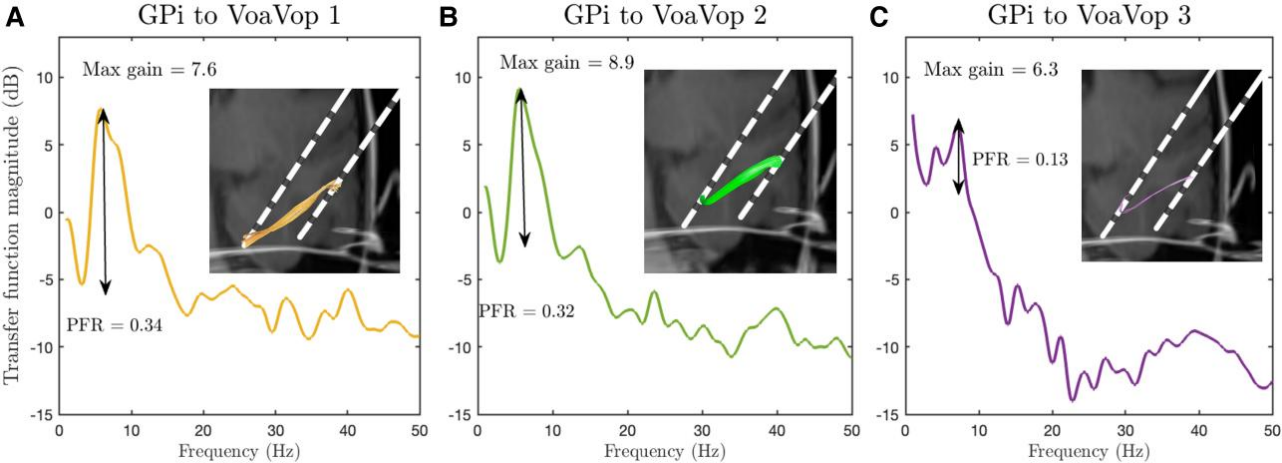
**An example of neural tracts.** This figure illustrates a sample of three unique neural pathways from GPi (left lead) to ventral oralis anterior-posterior (VoaVop) (right lead) for one patient, each characterized by varying FA, Ad, length (*L*), and number of fibres (*N*). Accompanying each pathway is its respective transfer function magnitude plot. Maximum gain and the peak-to-floor ratio (PFR) are annotated on each plot for clarity. The larger fibres represented in (**A**) (FA = 0.40, Ad = 1.35 mm^2^/s, *L* = 16.5 mm, total *N* = 3) and (**B**) (FA = 0.42, Ad = 1.38 mm^2^/s, *L* = 18.8 mm, total *N* = 132) exhibit higher maximum gain and PFR values. Conversely, the pathway depicted in (**C**) (FA = 0.38, Ad = 1.2 mm^2^/s, *L* = 20.07 mm, total *N* = 45), characterized by smaller FA and Ad has lower maximum gain and PF ratio, underscoring the relationship between the structural characteristics and functional connectivity. Ad, Axial diffusivity; FA, Functional anisotropy; *L*, Fibre length; *N*, Number of tracts.

**Figure 4:**
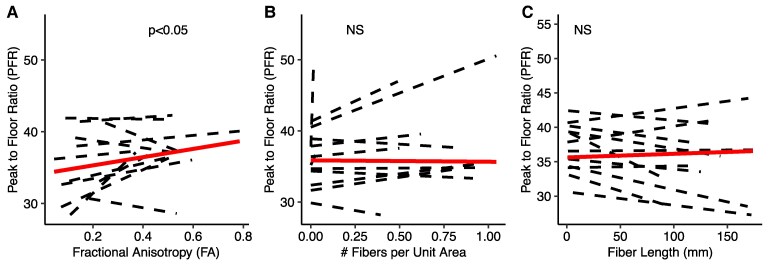
**Peak to floor ratio relationship with tractography measures.** Illustration of the linear fits by maximum likelihood and the statistical significance between the peak-to-floor (PFR) ratio and tractography measures: (**A**) FA, (**B**) number of fibres per unit area (*N*), and (**C**) fibre length (*L*). Individual subject PFRs are depicted by dashed lines.The solid lines represent the linear fit for group analysis. The *t*-test was performed on the estimates calculated from the linear mixed-effects models and the *P*-values of the *t*-statistic *P*-values were Bonferroni-corrected. Figure A highlights the significant correlation (*t*-value = 2.534, *P*-value < 0.01, Number of observations = 880, *N*_pid_ = 12) of PFR with FA. (**B**) and (**C**) shows the absence of significant correlation (NS) of PFR with *N* and *L*, respectively.

**Figure 5:**
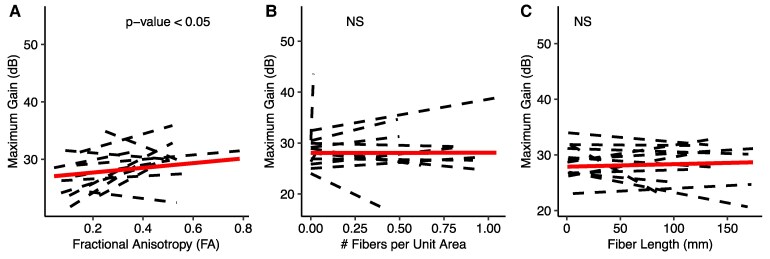
**Maximum gain relationship with tractography measures.** Illustration of the linear fits by maximum likelihood and the statistical significance between the maximum gains and tractography measures: (**A**) FA, (**B**) number of fibres per unit area (*N*), and (**C**) Fibre length (*L*). Individual subject maximum gains are depicted by dashed lines. The solid lines represent the linear fit for group analysis. The *t*-test was performed on the estimates calculated from the linear mixed-effects models and the *P*-values of the *t*-statistic *P*-values were Bonferroni-corrected. (**A**) Highlights the significant correlation (*t*-value = 2.663, *P*-value < 0.01, Number of observations = 880, *N*_pid_ = 12) of maximum gain with FA. (**B**) and (**C**) shows the absence of a correlation of maximum gain with *N* and *L*, respectively.

For clarification, we visualized three distinct pathways from the GPi to VoaVop in a single patient in Fig. 3, exemplifying the individual fibre tracts and their corresponding PF ratio and maximum gains. Evaluation of the relationship between the PF ratio (*R*^2^ = 0.25, *N*_pid_ = 12, number of observations = 880) and maximum gain (*R*^2^ = 0.13, *N*_pid_ = 12, number of observations = 880) with FA, *N*, and *L* indicated a significant positive correlation between both the PF ratio and maximum gain with FA and Ad, as illustrated in Figs. 4 and 5. Conversely, the analysis showed no statistically significant correlation between PF ratio and maximum gain with either fibre length (*L*) or the number of fibres per unit area (*N*) (Figs. 4 and 5). The results of the LME analysis are presented in Table 2, where we detail the estimated effects, 95% confidence intervals, and statistical significance of each predictor.

**Table 2:** Statistical results

DTI measures	Intercept [CI]	FA [CI]	*N* [CI]	L [CI]	*R* ^2^
**PF ratio**	**34.63** [32.13, 37.13]	**6.12** [1.38, 10.86]	1.44 [−1.10, 3.98]	−0.01 [−0.02, 0.00]	.25 (*N*_pid_ = 12)
**Maximum Gain**	**26.31** [24.17, 28.44]	**6.77** [1.78, 11.76]	1.19 [−1.52, 3.89]	0.0 [−0.02, 0.01]	.13 (*N*_pid_ = 12)

Statistical outcomes of estimated effects with 95% confidence interval (CI) from the linear mixed effect models fit for peak-to-floor ratio (PFR) and maximum gain in relation to FA, number of tracts (*N*), and fibre length (*L*) for 12 number of patients (*N*_pid_). The significant effects are highlighted with bold text.

## Discussion

Several research studies to date focused on integrating structural and functional connectivity in order to understand how they correlate and the mutual information they share with one another.^42-49^ These investigations predominantly depend on modalities such as electro-encephalography (EEG) and fMRI for assessing functional connectivity, while employing DTI to assess structural connectivity. However, despite significant insights offered by these studies, this relationship is still poorly understood. Notably, limitations such as the relatively low spatial resolution of EEG and the low temporal resolution of fMRI prevents us from conducting precise localization and capturing rapid neural dynamics while assessing functional connectivity.^50,51^ Thus, it seems necessary to employ other modalities to fully address the relationship between structural and functional connectivity in the brain.

In this paper, we aimed to study the relationship between DTI parameters and characteristics of patient-specific transfer function models obtained from deep brain intrinsic neural activity. Thus, we investigated the correlation between FA, Ad, Number of fibre tracts per unit area (*N*), and fibre tract length (*L*) as DTI parameters with maximum transfer function gain and PF ratio.

Our results, consistent with previous works done on the relationship between the cortical functional and anatomical connectivity^42-49^ provide further evidence of the relationship between anatomical and functional connectivity in deep brain regions. In particular, our results highlight positive correlation between FA and Ad with both characteristic of transfer function models (i.e. PF ratio and maximum gain).

Significant positive correlation between FA and maximum transfer function gain shows that axonal fibres with higher integrity can better amplify and spread the intrinsic brain signals throughout the brain network. FA significant positive correlation with PF ratio suggests that axonal fibres with higher white matter integrity have the capacity to provide larger system response and signal transmission.

Significant positive correlation of maximum transfer function gain and PF ratio with Ad suggests that the maximum level of signal amplification and fast magnitude drop-offs happen with higher magnitudes of diffusion parallel to fibre tracts. The lack of correlation between TF and the number of fibre tracts could be because tracts with larger fibres (or higher Ad) have a smaller number of fibres. One other possible conclusion from lack of correlation with the number of tracts is that functional connectomic models based on DTI may better reflect true functional signal transmission if they are based on FA and Ad rather than number of fibres.

In addition, Abe *et al*.^21^ previously provided evidence on the correlation between DTI parameters (i.e. FA, tract length, and tract diameter) and characteristics of DBS evoked potentials.^21^ Our previous results^21^ suggest that the integrity of white matter tracts plays a crucial role in determining the efficiency, strength, and transmission speed of DBS-induced signals. Moreover, we have demonstrated through a patient-specific transfer function approach that DBS pulses travel along normal pathways from the stimulation site to distant targets in the brain.^20^ These studies collectively highlight the varied role of DTI parameters, such as FA and Ad, not only in providing insights into anatomical connectivity, but also in explaining information transmission in the brain. These results can be significant for using DTI to create approximate whole-brain dynamic models. Future studies will focus on examining the relationship between the DTI parameters and transfer function phase delays. Another important implication of these results is to further validate the use of FA as a measure of clinical injury that not only reflects anatomical disruption of fibres but is likely to be related to decreased functional signal transmission, however, further research is needed.

## Limitation

As a non-invasive imaging technique, DTI provides valuable information about the white matter micro-structure which enables their visualization in 3D and facilitates studies on brain injuries. However, similar to any imaging technique it has certain limitations. First, such computations of anatomical connectivity rely on the orientation of fibres which might result in inaccuracies in regions where the fibres interactions are complex.^52^ This could be addressed by employing methods that do not suffer from crossing fibres inaccuracies, such as neurite orientation dispersion and density imaging techniques^53^ in future studies. Second, DTI has low image resolution, limiting its ability to identify single nerve fibres or small fibre bundles. Moreover, it is sensitive to noise and artifacts; therefore, we require precise motion control and post-processing approaches. To better utilize DTI, integration with other imaging techniques and clinical data is necessary.^52^ This is why we, previously, used DBS measurements to validate and confirm the effectiveness and accuracy of our DTI measurements for clinical applications.^21^ Moreover, our analysis of FA and Ad provided insights into tract-specific integrity, the choice of diffusion metrics warrants further discussion, particularly regarding the role of mean diffusivity.

In addition, it is crucial to acknowledge the limitations associated with Linear Time-Invariant (LTI) models, including transfer function analysis, which we employed to model the signal transmission between different nuclei in the deep brain regions. LTI models may not fully and accurately capture the dynamic and non-linear nature of brain function. The brain's complex and adaptive nature might involve time-varying dynamics that cannot be adequately addressed by LTI models.

Moreover, the transfer function gains here do not indicate that the information is transmitted through a direct pathway from input to the output of the pathway's system. Such measure can also indicate a common input to both system's input and output (two ends of a pathway), whether there is a fibre between them or not. However, in this study, since we are investigating the correlation of anatomical connectivity with the functional connectivity, there always exists a tract between the input and output. Therefore, this concern is not valid here, although it is a valid concern in general.

## Conclusion

In conclusion, our study provides further evidence for the existence of a significant relationship between structural and functional connectivity in the deep brain regions in children with dystonia, offering valuable insights into how white matter integrity affects intrinsic neural activity propagation in the brain network.

## Data Availability

The data is not publicly available as it contains patient information. However, it will be provided upon request, subject to approval through the required hospital screening process in compliance with HIPAA regulations. The code is available upon request.
